# Careful indication of the indwelling urinary catheter: impact on catheter-associated urinary tract infection rates

**DOI:** 10.1186/2047-2994-4-S1-P218

**Published:** 2015-06-16

**Authors:** MG Menegueti, AM Laus, M Auxiliadora-Martins, GG Gaspar, ML Puga, CA Feliciano, A Basile-Filho, F Bellissimo-Rodrigues

**Affiliations:** 1 HCFMRP/USP, Brazil; 2 EERP/USP, Brazil; 3 FMRP/USP, Brazil

## Introduction

Catheter-associated urinary tract infection (CAUTI) among critically ill patients and can provoke a high incidence of morbidity and mortality

## Objectives

Evaluate the impact of the implementation of a checklist to evaluate the use of indwelling urinary catheter (IUC) in the occurrence of CAUTI in critically ill patients of a tertiary university hospital

## Methods

This is a prospective study before-and-after that evaluated the IUC utilization rates and the occurrence of CAUTI after the implementation of training. The training consisted of early removal of this device, as well as everyday completing a checklist to review the need for IUC. The study was carried out from 2005 to 2010 (pre intervention) and from 2011 to 2014 (post intervention). Daily infection control committee (ICC) performed surveillance of CAUTI according to the criteria of (CDC / NHSN). The Student t test was applied to compare average of IUC utilization rates and the rates of CAUTI.

## Results

The utilization rates of this device in pre intervention period were 70, 75, 84, 72, 71 and 71%, respectively with an average of 74%. In the post-intervention period utilization rates were 60, 65, 54 and 49, with an average of 57%. We could observe a significant reduction in the use of IUC in critically ill patients of about 17%, p = 0.0024. Before using the checklist (pre-intervention) CAUTI rates had an average of 9.63 per 1,000 catheters-day, and the post intervention period the average was 3.58, with p = 0.005. We emphasize that throughout the study (pre and post intervention) CAUTI prevention measures were performed as: use of aseptic technique for catheter insertion; 2 accomplishment of catheter insertion and maintenance by educated personnel only; 3) emphasis on handwashing before and after manipulation of the system; 4) adequate catheter fixation; 5) maintenance of closed drainage system

## Conclusion

The use of daily check list for indication and maintenance of IUC in critically ill patients may be effective in reducing the utilization rate of this device, and consequently the reduction of CAUTI

## Disclosure of interest

None declared.

